# Investigation of the effects of some processing conditions on the fate of oxytetracycline and tylosin antibiotics in the making of commonly consumed cheeses from the East Mediterranean

**DOI:** 10.14202/vetworld.2021.1644-1649

**Published:** 2021-06-25

**Authors:** Hussein F. Hassan, Liz Saidy, Rita Haddad, Chadi Hosri, Shady Asmar, Adla Jammoul, Rola Jammoul, Hamad Hassan, Mireille Serhan

**Affiliations:** 1 Department of Natural Sciences, School of Arts and Sciences, Lebanese American University, Lebanon; 2 Department of Veterinary Sciences, Faculty of Agronomy, Lebanese University, Dekwaneh, Lebanon; 3 Research and Development Department, Dairy Khoury, Metn, Lebanon; 4 Lebanese Agricultural Research Institute, Fanar, Lebanon; 5 Faculty of Public Health, Lebanese University, Beirut, Lebanon; 6 Minister’s Office, Ministry of Public Health, Beirut, Lebanon; 7 Department of Nutritional Sciences, Faculty of Health Sciences, University of Balamand, Koura, Lebanon

**Keywords:** antibiotics, cheeses, liquid chromatography–mass-spectrometry, oxytetracycline, processing, tylosin

## Abstract

**Background and Aim:**

Transfer of antibiotics from raw milk to derived products is directly related to the processes involved in the manufacturing of dairy products, including East Mediterranean cheeses, since these have particular flow diagrams of production. The aim of this study was to assess the effects of skimming, pasteurization, curding, pressing, salting, cheese boiling, and whey acidification/heating on two widely used antibiotics in Lebanon, oxytetracycline (OTC) and tylosin (TYL), in the manufacture of commonly consumed cheeses in the East Mediterranean.

**Materials and Methods:**

Four hundred and fifty kilograms of full-fat bovine milk were spiked with OTC and TYL, then skimmed and pasteurized using holder and high-temperature short-time (HTST) methods. Milk was then processed to make cheeses (23 kg Baladi, 20 kg Akkawi, 20 kg Halloum, and 18 kg Double Cream). Liquid chromatography–mass-spectrometry was used to measure antibiotics. Analysis was performed using Statistical Package for the Social Sciences v25.

**Results:**

Skimming significantly (p=0.015) decreased TYL concentration by 68.6%. OTC degradation during holder (41-54%) proved to be significant (p=0.015). HTST had a significant (p=0.012) effect on TYL with 32% degradation. Curding step in making Baladi had a significant (p=0.028) effect on OTC only with the concentration increasing by 1.5-fold. Acidification and heating of whey to produce Double Cream decreased significantly (p=0.037) OTC concentration (14.7-46.3%), while TYL concentration increased significantly (p=0.000) by 300%. Pressing and salting in making Akkawi did not have any significant effect, while cheese boiling in making Halloum significantly decreased both antibiotics.

**Conclusion:**

OTC is transferred to Baladi and Akkawi (curd based) mainly, while double cream (whey based) has a high level of TYL transfer. Hence, people who consume these cheeses excessively could be exposed to high amounts of both antibiotics and thus be prone to their detrimental effect on health.

## Introduction

Antibiotics are used in both humans and animals for the treatment and prevention of several diseases caused by infectious agents. Health care and veterinary fields have been relying on them since the discovery of the first antibiotic in the 1940s [1]. Over the past decade, antibiotic consumption has increased dramatically [2], and with that, antibiotic resistance emerged [3]. Greater consumption of antibiotics was more evident in developing countries than in developed ones [3]. In Lebanon, the use of antibiotics has been emerging for medical use in humans as well as in the veterinary field. Worldwide, more than 50% of all antibiotics made are used in animal agriculture applications [4]. The present and foreseen development of the total population calls for expanding high-quality animal protein production. Dairy farming is considered as one of the most important ways of fulfilling this need, particularly in developing countries [1]. Dairy products display large nutritional, compositional, and cultural inconsistencies [5]. The East Mediterranean region has its unique white cheese selection such as Akkawi, Double Cream, and Halloum, among others, which are deemed part of a healthy diet as an abundant source of protein, calcium, and other essential nutrients [5]. In addition, in the East Mediterranean, there is an ascending trend of the proportion of energy intake from milk and dairy products [6]. In Lebanon, milk and dairy products provide 10% of total daily energy intake [7]. Nevertheless, this intake of dairy products is accompanied by exposure to antibiotics which can be divided into five categories according to their mode of action and spectrum of activity [8]. Oxytetracycline (OTC) and tylosin (TYL) belong to tetracycline and macrolide families, respectively. Tetracyclines are broad-spectrum antibiotics that act by targeting the ribosome of the bacteria. They are characterized by their exceptional chemotherapeutic efficiency to treat a wide range of microorganisms [9]. Their use has been associated with gastrointestinal disturbance, photosensitivity, and hepatotoxicity. Furthermore, they have a strong affinity to calcium and can accumulate in developing teeth and bones, leading to discoloration of teeth and inhibition of bone growth [10]. On the other hand, macrolides work by inhibiting the synthesis of bacterial proteins. They are recycled into bile by the liver and thus tend to accumulate in the body. Macrolides may produce inflammation and clinicians generally advise dispensing low doses of this drug [11]. Lots of parameters influence the degradation of antibiotics, such as the food matrix, methods of thermal processing, and presence of food additives [11]. Although the precise impact is still unclear, the food matrix has been proven to influence the degradation of antibiotic residues. There lies high importance in characterizing and understanding how antibiotics are partitioned in milk and dairy products to determine the consumers’ potential exposure levels. Doing so by understanding the drivers of the distribution and concentration of antibiotic residues among milk fractions will enhance and clarify the potential risk assessment for human exposure [10,12].

The scientific literature regarding the transfer of antibiotics from raw milk to milk products is limited. Furthermore, this transfer is directly related to the processes involved in the manufacturing of dairy products. Thus, investigating the degradation patterns of antibiotics during processing is of prime importance, especially among East Mediterranean cheeses, since these have particular flow diagrams of production.

This study aimed to investigate the effects of heating, rennet addition, pressing, acidification, skimming, and salting which were assessed. Since the local authorities do not have any standard when it comes to antibiotic residues in East Mediterranean cheeses, our work will help in understanding the patterns of destruction and partitioning in these widely consumed cheeses, and thus, in developing the maximum residue levels.

## Materials and Methods

### Ethical approval and Informed consent

Approval of the Institutional Review Board at the Lebanese American University was obtained before approaching the veterinarians.

### Study period and location

This study was carried out from June 2019 to June 2020. Inoculating milk with antibiotics and processing it into different products was done at Dairy Khoury, a major dairy industry in Lebanon, so that our results reflect the actual parameters used by the industry. For the analytical work, the laboratory of the Lebanese Agriculture Research Institute (Metn, Mount Lebanon) was chosen since it is officially accredited by the Lebanese government for the detection and quantification of antibiotic residues in food, in addition to it being ISO17025 certified.

### Choice of the antibiotics

A short phone-based questionnaire was conducted by the study investigators among all veterinarians employed by dairy farms in Lebanon through a database obtained from the Lebanese Ministry of Agriculture. It showed that TYL and OTC were among the four most commonly used antibiotics for cows in Lebanon.

### Milk inoculation and cheese manufacturing

Four hundred fifty kg of antibiotic-free bovine milk was inoculated with 75 mL Inj. Oxytetravet 30% (Mobedco, Saudi Arabia) and 112.5 mL of Tylokel (ELA N.V., Belgium). Then, the spiked milk was divided into three batches (A, B, and C), whereby each batch has undergone different processing conditions. One hundred kilograms of spiked milk A have undergone holder pasteurization (63°C for 30 min), while 50 kg of B was centrifuged at 3500 rpm for skimming, then mixed with another 50 kg of spiked full-fat milk. This is the practice performed in the local dairy industries to get better organoleptic quality in the resulting dairy products. After that, the resultant milk mixture has undergone holder pasteurization (63°C for 30 min). Finally, 250 kg of batch C underwent high-temperature short-time (HTST) pasteurization (72°C for 15 s). The three batches of pasteurized milk were then processed to obtain different cheeses.

Microbial rennet (CHY-MAX® Powder Extra NB, Christian Hansen, Denmark) was then added to the pasteurized milk (at 40°C), then mixed and left to rest for 25 min. Subsequently, the solid curd was collected by filtration, and the liquid whey was separated and collected. Curd was drained and shaped in molds to obtain the “Baladi” (cottage) cheese. Then, Baladi cheese has undergone pressing using a mechanical pressor, and the resulting cheese was the “Akkawi.” Akkawi cheeses were dropped in boiling water and left until the core temperature reached 70°C to get the “Halloum” cheese, which was then salted in 5% brine solution to yield 2% salt Halloum and in 12% brine solution to yield 6% salt Halloum. Whey that was collected in the curding step was heated to 88°C in the presence of salt and citric acid, leading to the clotting of the whey proteins. These proteins were collected and pressed to get Double Cream cheese.

### Sample extraction

Liquid-liquid extraction was developed and verified in the laboratory from the official European Union method to be able to extract the antibiotics from the sample without the interference of the coextracts [13]. It involves weighing 2±0.05 g portion of the dairy product. Then, for OTC extraction, 200 μL of formic acid 0.1% were added, followed by 1 min shacking, then left to rest for 10 min in obscurity. Then, 0.5 mL Na_2_ EDTA was added and the mixture was shaken for 1 min, after which addition of 8 mL of 5% TCA was done, followed by 10 min mixing. Next, the samples were centrifuged for 10 min at 10,000× relative centrifugal force (RCF) at 4°C, and the extracts were filtered through 0.22 μm polyvinylidene fluoride (PVDF) filters into amber vials. As for TYL extraction, 2g±0.05 g of the sample was weighed, and 200 μL of formic acid 0.1% was added and followed by shaking for 1 min and left in obscurity for 10 min. Then, 10 mL of acetonitrile was added to the solution, followed by shaking for 5 min. The next step was to centrifuge for 10 min at 10,000× RCF at 4°C. Then, the extracts were filtered through 0.22 μm PVDF filters into amber vials [13].

### Instrumental analysis by Liquid chromatography–mass-spectrometry (LCMS)

LCMS (SCHIMADZU LCMS-8045) with HPLC (Nexera X2 LC-30AD Liquid Chromatography, Degassing unit: DGU-20A5R, Communication Bus Mobile CBM-20A, and Prominence Column Oven CTO-20AC) was used. The mass spectrometer was operated in the positive ESI ion mode. Nitrogen was used as nebulizer gas, curtain gas, and collision gas. The mass spectrometer temperature was 300°C. The chromatographic separation was performed on a 2.1×100 mm, 3 µm column. The elution was performed in a gradient mode. An in-house validation protocol was carried out to establish the performance characteristics of the method, ensuring adequate identification, confirmation, and quantification of OTC and TYL. The method was validated in milk, whey, and cheese. The selectivity and specificity were assessed by analyzing three blank samples from each matrix. The absence of background peaks, above a signal-to-noise ratio of 10, at the retention time of OTC showed that the method was free of endogenous interferences. The validity was determined by analyzing three separate samples of milk that were each spiked with OTC at a level of 100 µg/L, equivalent to the MRL of OTC in milk. The obtained trueness expressed as the percentage of recovery was 105% for OTC and TYL was 100% for milk. The limit of quantification (LOQ) was defined as the lowest concentration or mass of the analyte that has been validated with acceptable accuracy, by applying the complete analytical method. The calculated LOQ was 10 mg/kg for milk. The linearity was determined through an analytical curve obtained through the LC–MS for each antibiotic. This was through six points covering a range of concentrations (100, 300, 500, 600, 800, 1000, and 1500 ppb). The parameter of this calibration curve showed good linearity, with a correlation coefficient >0.995.

### Statistical analysis

Analysis was performed using Statistical Package for the Social Sciences version 25.0 (IBM Corp., NY, USA). Descriptive analysis was used to summarize the study variables and to screen for out of range values. Continuous variables were described using mean and standard deviations. Shapiro–Wilk was used to assess data normality. Paired t-tests were used to compare the mean differences in OTC and TYL values within groups for the effect of holder heating, curding, pressing, cheese boiling, salting, and whey processing. Two-tailed p-values are reported. p<0.05 was considered statistically significant.

## Results and Discussion

In this study, we assessed the effects of skimming, pasteurization, curding, pressing, salting, cheese boiling, and whey acidification/heating on two widely used antibiotics in Lebanon, OTC and TYL, in the manufacture of commonly consumed cheeses in the East Mediterranean. OTC and TYL molecules were non-homogenously distributed between the fractions (whey, Baladi, Akkawi, Halloum, and Double Cream). For OTC, mean concentration factors of 1.8±0.3 and 1.7±0.2 from heat-treated milk to Baladi (curd) and Akkawi, respectively, were calculated. On the other hand, this factor was calculated as 0.2 for Double Cream cheese made from whey. The highest concentrations among dairy products were reported at a range of 20.0-33.0 ppm and 22.9-28.0 ppm, in Baladi and Akkawi, respectively. For TYL, mean concentration factors were calculated as 1.25±0.2 and 2.1±0.5 between heat-treated milk and curd (Baladi), and between whey and Double Cream, respectively. Double Cream had the highest concentration of TYL, ranging between 7.1 and 8.6 ppm, being the only cheese that had a level of TYL higher than that of the raw spiked milk (7.8 ppm).

### Effect of pasteurization

Milk was subjected to the two common pasteurization techniques in the East Mediterranean (holder and HTST pasteurization). Subjecting milk to holder pasteurization decreased significantly (p=0.015) OTC concentration by 41-54%; while HTST led to a non-significant 18% decrease in the OTC concentration (p=0.523) (Table-1). As for TYL, holder pasteurization had no significant effect (p=0.561), and the decrease was 21-32%; whereas HTST decreased (32%) significantly TYL concentration (p=0.012) (Table-2).

**Table-1:** Effect of different processes on OTC concentration.

Process	OTC			

	Before (ppm)	After (ppm)	p-value	
Holder heating	24.5	14.5	0.015*	
	26.0	12.0		
HTST heating	24.5	20.0	0.523	
Skimming	24.5	20.0	0.411	
Curding	14.5	31.0	0.028*	
	12.0	20.0		
	20.0	33.0		
Pressing	31.0	28.0	0.526	
	20.0	22.9		
	33.0	24.0		
Cheese boiling	28.0	11.4	0.031*	
	22.9	10.6		
	
Salting	**0%**	**2%**	**6%**	**p-value**
	
	11.4	11.5	15.0	0.500
	10.6	9.9	11.0	
		10.0	9.5	
	
Whey processing	**Before (ppm)**	**After (ppm)**	**p-value**	
	
	3.5	3.0	0.037*	
	6.0	3.4		
	6.0	3.2		

* p<0.05. OTC=Oxytetracycline, HTST=High-temperature short-time

**Table-2:** Effect of different processes on TYL concentration.

Process	TYL			

	Before (ppm)	After (ppm)	p-value	
Holder heating	7.8	5.3	0.561	
	3.9	3.1		
HTST heating	7.8	5.3	0.012*	
Skimming	7.8	3.0	0.015*	
Curding	3.1	4.4	0.575	
	5.3	5.7		
Pressing	4.4	5.5	0.954	
	5.7	4.7		
Boiling cheese	5.0	1.6	0.007*	
	5.5	3.1		
	4.7	2.8		
	
Salting	**0%**	**2%**	**6%**	**p-value**
	
	1.6	2.1	2.0	1.000
	3.1	3.2	2.8	
	2.8	2.2	2.6	
	
	**Before (ppm)**	**After (ppm)**	**p-value**	
	
Whey processing	2.8	8.2	0.000*	
	2.9	8.6		
	2.5	7.1		

* p<0.05. TYL=Tylosin, HTST=High-temperature short-time

Many studies reported results addressing the effect of heat processing on antibiotic residues, including OTC with a destruction rate ranging between 25 and 100% [11,14-18]. However, only a few studies addressed this effect in the milk matrix [19]. Our study reported a significant effect (41-54% decrease) of Holder pasteurization on OTC (p=0.015). Other studies looking at the effect of holder pasteurization on OTC in milk did not report a significant effect with degradation levels of 10% as reported by Gajda *et al*. [20] and 15.3% as reported by Kellnerová *et al*. [19]. This could be attributed to the differences in experimental set-ups since our study was done in an industrial setting. As for HTST, no study in the literature reported its effect on OTC. Our results suggest sensitivity to thermal process time rather than temperature when it comes to OTC degradation.

As for TYL, studies looking at the effect of heat treatment on this antibiotic are scarce. Only one study looked at the effect of different heat treatments on TYL in milk using antimicrobial essays. This study found 51% decrease in TYL concentration after heating the milk at 120°C for 20 min, 21% decrease when heated at 140°C for 10 s, and an even lower reduction when exposed to holder pasteurization [21]. Similarly, our results reported an insignificant effect (21-32% decrease) of holder pasteurization and a significant effect (32% decrease) of HTST treatment.

### Effect of skimming

Skimming decreased milk fat concentration from 3.5% to 0.1% (Table-3). It did not significantly (p=0.411) decrease (18%) the OTC concentration (Table-1); while for TYL, it caused a significant (p=0.015) decrease by 62% (Table-2). Our results for OTC are in line with what [22] reported since OTC is highly hydrophilic. As for TYL, it was recovered mostly in the cream of the milk, indicating a high affinity to fat. No previous studies in the literature looked at the effect of skimming on TYL.

**Table-3:** Mean fat composition (±standard deviation) of raw and skimmed milk.

Milk	Fat (%)	

	Mean	SD
Raw	3.47	0.03
Skimmed raw	0.08	0.02

SD=Standard deviation

### Effect of curding

Curding resulted in a significant (p=0.028) increase (65-114%) in OTC concentration (Table-1); while the concentration increase was not significant (p=0.575) for TYL (Table-2).

Curding caused a significant increase in OTC concentration from heat-treated milk to Baladi by 1.4-fold. Similarly, this increase of OTC from milk to curd (Baladi) was reported by [10] (by 4-fold), [10] (by 3-5-fold), and [12] (by 2-fold). Our results propose a high affinity and interaction between OTC and the casein part of the milk. These results are in line with what [10] reported in terms of the OTC’s binding to animal proteins and its very high affinity to Ca^2+^ and Mg^2+^. In this regard, 80% of milk proteins are casein, and thus, they are mainly present in the curd fraction [23], and conceivably, OTC becomes bound to and tracks along. Since OTC was found to be concentrated in Baladi and Akkawi, special attention must be given when it comes to the dietary exposure of OTC from consuming these two products compared to other cheeses such as Halloum and Double Cream.

In contrast to OTC, curding step caused a non-significant increase in TYL concentration from milk to Baladi (curd). This could be attributed to its low affinity to casein proteins. This goes in line with [24-26] who reported that TYL was found to bind to casein at a rate of 15% only.

### Effect of pressing, cheese boiling, and salting

Pressing was accompanied by a slight, yet not significant, decrease of 2-6% in moisture content from Baladi to Akkawi (Table-4). Pressing Baladi to make Akkawi had no significant effect on the concentration of both OTC (p=0.526) (Table-1) and TYL (p=0.954) (Table-2).

**Table-4:** Mean moisture content (±standard deviation) of Baladi, Akkawi, and Halloum from the three treatments (A, B, and C).

Product	Moisture (%)	

	Mean	SD
C – Halloum cheese 0%	45.4	0.8
A – Halloum cheese 2%	53.8	1.2
B – Halloum cheese 2%	54.4	2.9
C – Halloum cheese 2%	55.8	0.9
A – Halloum cheese 6%	46.8	1.2
B – Halloum cheese 6%	48.3	1.2
C – Halloum cheese 6%	46.8	1.5
A – Baladi cheese	63.1	1.9
B – Baladi cheese	63.0	1.3
C – Baladi cheese	61.4	1.1
A – Akkawi cheese	56.9	0.3
B – Akkawi cheese	61.2	0.7
C – Akkawi cheese	58.7	0.3

SD=Standard deviation

Boiling Akkawi to make Halloum cheese caused a significant decrease of OTC (p=0.031) by 54-59% (Table-1) and of TYL (p=0.007) by 40-68% (Table-2). The effect of boiling Akkawi to obtain Halloum reported in our study was significant (p=0.007). This process involves reaching a core temperature of 70°C (for a duration of approximately 35 min). As previously mentioned, studies in the literature and our results support that these conditions cause a significant level of destruction of TYL. Hence, in the case of Halloum making, the significant decrease in the levels of TYL makes it safer to consume.

Salting Halloum to reach a salt content of 2 and 6% did not significantly alter OTC (Table-1) and TYL (Table-2) concentration. This was also accompanied with slight changes in moisture content (Table-3). No study in the literature assessed the effect of these three aforementioned studies.

### Effect of heating and acidification of the whey

Whey processing to make Double Cream significantly decreased OTC (14-47 %) (p=0.037) (Table-1); while it significantly increased TYL concentration (184-197%) (p=0.000) (Table-2). This might suggest that contrary to OTC, TYL might have a high affinity to whey proteins. TYL was found to be concentrated in Double Cream, making this product less safe when it comes to TYL exposure from its consumption. This points out the need for regulations regarding cheeses and antibiotic levels and enforcing safety limits. Moreover, our results indicate that cheeses produced from the whey fraction might be of higher risk due to TYL levels, while those made from the curd seem to be safer to consume in this regard.

## Conclusion

Looking at antibiotic residues in milk and milk products is important from a health and processing point of view. Our first-of-its-kind study on East Mediterranean cheeses proved that OTC is significantly affected by holder pasteurization, but not with HTST, while TYL is significantly affected by HTST, but not holder pasteurization. As for milk derivatives, OTC is transferred from spiked milk to cheeses, especially Baladi and Akkawi, making them higher risk products for this antibiotic compared to Halloum and Double Cream. In contrast, Double Cream had a high level of TYL transfer, while the other cheeses did not. Hence, it is important to highlight that people who consume excessively these products, in which OTC and TYL might become concentrated, could be exposed to significant amounts of both antibiotics. In conclusion, it is essential to correctly employ control method throughout the whole milk production chain to avoid any possible risk that the presence of antibiotics might cause.

## Authors’ Contributions

HFH and MS: Designed the study, secured the funding, supervised the data collection, and wrote the manuscript. LS and RH: Carried the laboratory work and statistical analysis. CH: Followed up on data collection from veterinarians; SA: Inoculated the milk and processed it to different cheeses. AJ and RJ: Standardized the LCMS and co-supervised the data collection. HH: Helped in statistical analysis and co-wrote the manuscript. All authors read and approved the final manuscript.
